# The vaginal microbiota of pregnant women who subsequently have spontaneous preterm labor and delivery and those with a normal delivery at term

**DOI:** 10.1186/2049-2618-2-18

**Published:** 2014-05-27

**Authors:** Roberto Romero, Sonia S Hassan, Pawel Gajer, Adi L Tarca, Douglas W Fadrosh, Janine Bieda, Piya Chaemsaithong, Jezid Miranda, Tinnakorn Chaiworapongsa, Jacques Ravel

**Affiliations:** 1 Perinatology Research Branch, Program for Perinatal Research and Obstetrics, Division of Intramural Research, Eunice Kennedy Shriver National Institute of Child Health and Human Development, NIH, Bethesda, MD, USA; 2 Department of Obstetrics and Gynecology, University of Michigan, 1500 East Medical Center Drive, Ann Arbor, MI 48109, USA; 3 Department of Epidemiology and Biostatistics, Michigan State University, Room B601, 909 Fee Road, East Lansing, MI 48824, USA; 4 Department of Obstetrics and Gynecology, Wayne State University School of Medicine, 540 E Canfield St, Detroit, MI 48201, USA; 5 Institute for Genome Sciences, University of Maryland School of Medicine, 801 West Baltimore Street, Baltimore, MD 21201, USA; 6 Department of Microbiology and Immunology, University of Maryland School of Medicine, 685 W Baltimore St #480, Baltimore, MD 21201, USA; 7 Hutzel Women’s Hospital, Detroit Medical Center, 3990 John R, Detroit, MI 48201, USA

**Keywords:** Infection-induced preterm delivery, Histologic chorioamnionitis, Prematurity, Vaginal flora, Vaginal microbiome

## Abstract

**Background:**

This study was undertaken to determine whether the vaginal microbiota of pregnant women who subsequently had a spontaneous preterm delivery is different from that of women who had a term delivery.

**Results:**

This was a nested case–control study of pregnant women who had a term delivery (controls) and those who had a spontaneous preterm delivery before 34 weeks of gestation (cases). Samples of vaginal fluid were collected longitudinally and stored at −70°C until assayed. A microbial survey using pyrosequencing of V1-V3 regions of 16S rRNA genes was performed. We tested the hypothesis of whether the relative abundance of individual microbial species (phylotypes) was different between women who had a term versus preterm delivery. A suite of bioinformatic and statistical tools, including linear mixed effects models and generalized estimating equations, was used. We show that: 1) the composition of the vaginal microbiota during normal pregnancy changed as a function of gestational age, with an increase in the relative abundance of four *Lactobacillus* spp., and decreased in anaerobe or strict-anaerobe microbial species as pregnancy progressed; 2) no bacterial taxa differed in relative abundance between women who had a spontaneous preterm delivery and those who delivered at term; and 3) no differences in the frequency of the vaginal community state types (CST I, III, IV-B) between women who delivered at term and those who delivered preterm were detected.

**Conclusions:**

The bacterial taxa composition and abundance of vaginal microbial communities, characterized with 16S rRNA gene sequence-based techniques, were not different in pregnant women who subsequently delivered a preterm neonate versus those who delivered at term.

## Background

Preterm delivery is the leading cause of perinatal morbidity and mortality worldwide [1-8]. There are approximately 15 million preterm births every year [1,3,8], and few approaches have been proven successful to reduce the rate of preterm birth and neonatal morbidity [9-11]. The cost of preterm birth to society has been estimated to be more than $26 billion per year in the United States alone [12-14]; therefore, the prediction and prevention of preterm birth is a major health care priority.

Of all preterm births, two-thirds occur after the spontaneous onset of preterm labor (with intact or ruptured membranes) [15]. Multiple mechanisms of disease have been implicated in the onset of spontaneous preterm labor (that is, infection/inflammation, uterine overdistension, decidual senescence, and so on). [16,17]. In normal pregnancy, the amniotic cavity is considered ‘sterile’; yet, microbial invasion of the amniotic cavity (MIAC), often subclinical in nature, occurs in one of every four preterm deliveries [18-20]. Microorganisms and their products can induce a local inflammatory response in gestational tissues (acute chorioamnionitis), leading to preterm labor [18-37]. Most intra-amniotic infections are thought to occur when microorganisms in the lower genital tract (vagina and/or cervix) gain access to the amniotic fluid [17]. Changes in the microbial ecosystem of the vagina have been implicated in the genesis of ascending intrauterine infection [17,20,31,38-45].

*Trichomonas vaginalis* infection [46-48] and bacterial vaginosis [28,41,43,49-67] are risk factors for spontaneous preterm labor and delivery; yet, identification of the patient with either of these conditions who will subsequently have a preterm delivery has proven difficult [60]. Characterization of the microbial composition of ecological niches in the human body [68-77], including the vagina, using culture-independent techniques, is now possible [78-96]. We previously reported a survey of the microbial communities of the vagina using sequencing of the 16S ribosomal RNA (rRNA) gene in normal pregnancy [97]. However, there is no information as to whether changes in the microbial composition of the vagina (using sequence-based techniques) occur prior to the onset of spontaneous preterm delivery.

The purpose of this study was to determine whether the longitudinal vaginal microbiota composition and structure of pregnant women who subsequently had a spontaneous preterm delivery is different from that of women who had a normal spontaneous term delivery. The major findings reported herein are that the vaginal microbiota changes with gestational age in women who deliver at term, and no differences were detected in the bacterial taxa, relative abundance and frequency of community state types between patients who deliver at term and those who subsequently had a spontaneous preterm delivery.

## Methods

### Study design

This was a nested case–control study conducted to compare changes in the vaginal microbiota of patients who had a spontaneous preterm labor and delivery with those who had an uncomplicated pregnancy. Cases and controls were selected in a 1:4 ratio from a prospective longitudinal cohort study designed to examine the relationship between biological markers and pregnancy outcome. The study included 18 cases and 72 controls. Patients with indicated preterm birth (for example, preeclampsia, intrauterine growth restriction, or congenital anomalies) were excluded. Patients volunteered to participate in the study and signed a written informed consent. The use of samples from the longitudinal study of pregnant women was approved by the Human Investigations Committee of Wayne State University and the Institutional Review Board (IRB) of the Eunice Kennedy Shriver National Institute of Child Health and Human Development (NICHD).

### Clinical definitions

A normal pregnant woman was defined as one without obstetrical, medical or surgical complications, who delivered at term (38 to 42 weeks) without congenital anomalies or acute histologic chorioamnionitis. Preterm labor was diagnosed by the presence of at least two uterine contractions every 10 minutes associated with cervical changes in patients with a gestational age between 20 and 34 weeks. Preterm premature rupture of membranes (PPROM) was identified with a sterile speculum examination with documentation of vaginal pooling and positive nitrazine and ferning tests. Spontaneous preterm delivery was defined as having occurred prior to the 34^th^ week of gestation in patients with either intact membranes or PPROM. Acute histologic chorioamnionitis was diagnosed based on the presence of inflammatory cells in the chorionic plate and/or chorioamniotic membranes [98-100]. Sixty-one percent (11/18) of the cases had evidence of acute histologic chorioamnionitis.

### Study procedures

Pregnant women who agreed to participate in the study had a speculum examination at each visit; a sample of vaginal fluid was collected under direct visualization from the posterior vaginal fornix by an obstetrician or midwife using a Dacron swab (medical packaging swab – PAK™ Carmarillo, CA, USA). The protocol called for sample collection every 4 weeks until 24 weeks of gestation, and then every 2 weeks until the last prenatal visit. Vaginal swabs were placed in a tube without any buffer and immediately stored at −70°C until assayed.

### DNA extraction, amplification and pyrosequencing of barcoded V1-V3 hypervariable regions of 16S rRNA genes

Procedures for the extraction of genomic DNA from frozen vaginal swabs have been developed and validated previously [101,95,102]. Briefly, frozen vaginal swabs were immersed in 1 ml of sterile PBS and vortexed for 10 minutes. A total of 500 μl of the cell suspension was mixed with 500 μl of pre-warmed (55°C) cell lysis buffer composed of 0.05 M potassium phosphate buffer containing 50 μl lyzosyme (10 mg/ml), 6 μl of mutanolysin (25,000 U/ml; Sigma-Aldrich, St. Louis, MO, USA) and 3 μl of lysostaphin (4,000 U/ml in sodium acetate; Sigma-Aldrich) and the mixture was incubated for one hour at 37°C. Then 10 μl proteinase K (20 mg/ml), 100 μl 10% SDS, and 20 μl RNase A (20 mg/ml) were added and the mixture was incubated for one hour at 55 °C. The samples were transferred to a FastPrep Lysing Matrix B tube (MP Biomedical, Santa Ana, CA, USA) and microbial cells were lysed by mechanical disruption using a bead beater (FastPrep instrument, Qbiogene, Carlsbad, CA, USA) set at 6.0 m/second for 30 seconds. The lysate was processed using the CellFree500 kit on a QIAsymphony robotic platform. The DNA was eluted into 100 μl of TE (10 mM Tris–HCl, 1 mM EDTA) buffer, pH 8.0. This procedure provided between 2.5 and 5 μg of high quality whole genomic DNA from vaginal swabs.

The bacterial species composition and abundance in vaginal communities were determined using culture-independent methods. The V1-V3 hypervariable regions of the 16S rRNA gene were amplified using an optimized primer set comprising 27 F [103] and 534R. Because primer 534R contains a unique sample identifying barcode, up to 192 samples were sequenced per run and generated 4,000 to 6,000 sequence reads per sample. The primers were as follows:

27 F - 5′-*GCCTTGCCAGCCCGCTCAG*TC**AGAGTTTGATCCTGGCTCAG**-3′

534R - 5′-*GCCTCCCTCGCGCCATCAG*NNNNNNNNCA**TTACCGCGGCTGCTGGCA***-***3**′

The italicized sequences are the 454 Life Sciences® primers B and A in 27 F and 534R, respectively, and the bold font denotes the universal 16S rRNA primers 27 F and 534R. The barcode within 534R is denoted by eight Ns (but varies from six to eight Ns) and were identical to those used by the Human Microbiome Project [104]. A mixture of bacterial 27 F primers was used to maximize sequence type discovery and eliminate the PCR amplification bias described by Frank *et al*. [103]. The 27 F formulation remains relatively simple: having only seven distinct primer sequences there is minimal loss of overall amplification efficiency and specificity. The 27 F primer mixture was: four parts of four-fold-degenerate primer 27f-YM (5′-AGAGTTTGATYMTGGCTCAG, where Y is C or T) plus one part each of primers specific for the amplification of *Bifidobacteriaceae* (27f-Bif, 5′-AGGGTTCGATTCTGGCTCAG), *Borrelia* (27f-Bor, 5′-AGAGTTTGATCC TGGCTTAG), and Chlamydiales (27f-Chl, 5′-AGAATTTGATCTTGGTTCAG) sequences. This primer formulation was previously shown to better maintain the original rRNA gene ratio of *Lactobacillus* spp. to *Gardnerella* spp. in quantitative PCR assays, particularly under stringent amplification conditions [103].

For every set of 192 vaginal genomic DNA samples, PCR amplification of 16S rRNA genes was performed in 96-well microtiter plates as follows: 1× PCR buffer, 0.3 μM primer 27 F and 534R, 0.25 μl HotStar HiFidelity DNA polymerase (5 U/μl; Qiagen, Germantown, MD), and 25 ng of template DNA in a total reaction volume of 25 μl. Reactions were set up on a QIAgility robotic platform in a semi-sterile environment. Reactions were run in a DNA engine Tetrad2 instrument (Bio-Rad, Hercules, CA) using the following cycling parameters: 5 minutes denaturing at 95°C followed by 29 cycles of 30 seconds at 94°C (denaturing), 30 seconds at 52°C (annealing) and 60 seconds at 72°C (elongation), with a final extension at 72°C for 10 minutes. Separate plates containing negative controls without a template for each of the 96 barcoded primers were included for each set of plates processed: in our workflow, if one of these samples is positive, the samples and negative controls plates are rerun with new primers; however, no amplicons were observed in any of the no template controls. The presence of amplicons was confirmed by gel electrophoresis on a 2% agarose gel stained with SYBRGreen (Life Technologies, Carlsbad, CA, USA). PCR products were quantified using the Quant-iT Picogreen® quantification system (Life Technologies) and equimolar amounts (100 ng) of the PCR amplicons were mixed in a single tube using the QIAgility robotic platform. Amplification primers and reaction buffer were removed by processing the amplicon mixture with the Agencourt AMPure Kit (Beckman-Coulter, Pasadena, CA, USA). All PCR amplification reactions that failed were repeated twice using different amounts of template DNA, and if these failed, the samples were excluded from the analysis.

The purified amplicon mixtures were sequenced by 454 pyrosequencing using 454 Life Sciences® (Roche/454 Life Sciences, Branford, CT) primer A by the Genomics Resource Center at the Institute for Genome Sciences, University of Maryland School of Medicine, using Roche/454 Titanium chemistries and protocols recommended by the manufacturer and amended by the Center.

All sequences were trimmed before the first ambiguous base pair. The QIIME software package [105] was used for quality control of the sequence reads using the split-library.pl script and the following criteria: 1) minimum and maximum length of 250 bp and 450 bp; 2) an average of q25 over a sliding window of 25 bp. If the read quality dropped below q25, it was trimmed at the first base pair of the window then reassessed for length criteria; 3) a perfect match to a barcode sequence; and 4) presence of the 534R 16S primer sequence used for amplification. Sequences were binned based on sample-specific barcode sequences and trimmed by removal of the barcode and primer sequences (forward, if present, and reverse). High-quality sequence reads were first de-replicated (99% similarity) using the UCLUST software package [106]. Detection of potential chimeric sequences was performed using the UCHIME component of UCLUST [107] with the *de novo* algorithm. Chimeric sequences were removed prior to taxonomic assignments. Taxonomic assignments were performed on each individual quality checked 16S rRNA sequence read using a combination of pplacer [108] and speciateIT (speciateIT.sourceforge.net). Taxonomic assignments (sequence read counts and relative abundances) are shown in Additional file 1: Table S1. All sequence data and metadata were deposited in the Sequence Read Archive (SRA; http://www.ncbi.nlm.nih.gov/Traces/sra/) under BioProject PRJNA242473 (SRA accession SRA150182, SRP040750).

### Statistical analysis

The abundance of bacteria is generally expressed on a logarithmic scale (base 10), given the wide range of bacterial abundance and the exponential nature of bacterial growth under certain circumstances (for example *in vitro*). The standard is to compare microbial abundance over time using the difference of logs, log_10_ (a) - log_10_ (b), which is the same as the log fold change log_10_ (a/b), where a and b are relative abundances of a given microorganism in two samples (for example, two sampling time points).

Changes in the abundance of a complex microbial ecosystem within the same patient at different time points were estimated for specific phylotypes. We assessed the dissimilarity between community states (in other words, how divergent community states are) using the Jensen-Shannon metric [109]. In microbial ecology, the term ‘community state’ refers to the relative abundance of all phylotypes at a particular time point in a subject; in our case, a sample of vaginal fluid.

The Jensen-Shannon divergence between two community states, p and q, is the average of the Kullback–Leibler divergences D_KL_ (p, a) and D_KL_ (q, a):

$$D_{\mathrm{JS}}\left(p,q\right)=\frac{D_{\mathrm{KL}}\left(p,a\right)+D_{\mathrm{KL}}\left(q,a\right)}{2}$$

where *a* is the mean of *p* and *q* and D_KL_(p,q) is the Kullback–Leibler divergence defined as:

$$D_{\mathrm{KL}}\left(p,q\right)=\sum_{i=1}^{n}P_{i}\log\left(\frac{P_{i}}{q_{i}}\right)$$

where p = (p_1_, …, p_n_) and q = (q_1_, …, q_n_).

The Kullback–Leibler divergence D_KL_(p, q) calculates the mean log fold changes log (p_i_/q_i_). While the Kullback–Leibler measure is widely used, it has one drawback: its value becomes infinite if one of the components of q is zero. In contrast, the Jensen-Shannon divergence always yields a value between 0 and 1. A Jensen-Shannon divergence score of 0 means that two community states are the same. In contrast, a Jensen-Shannon divergence score of 1 means that the two community states are completely different. The square root of the Jensen-Shannon divergence is called ‘Jensen-Shannon distance.’

The term ‘community state type’ is used in microbial ecology to describe a group of community states with similar microbial phylotype composition and abundance [95,110]. Such grouping is desirable in order to reduce dimensionality. Utilizing Jensen-Shannon divergence as a measure of dissimilarity among community states and hierarchical clustering with Ward linkage, six vaginal community state types in pregnant and non-pregnant women have been previously identified [95,97]. Four of the community state types (I, II, III and V) are dominated by *Lactobacillus* spp. (*Lactobacillus crispatus, L. gasseri*, *L. iners*, and *L. jensenii*, respectively) and the remaining two community state types (IV-A, IV-B) consist of microbial ecosystems with a diverse array of anaerobes and strict anaerobes, and substantially lower numbers of *Lactobacillus* spp. than the other community state types.

### Statistical procedures to evaluate the differential abundance of phylotypes between women who deliver at term and those who had a spontaneous preterm delivery

In order to assess a change in phylotype relative abundance between the two groups, we modeled the relative abundance of one phylotype at a time as a function of study group (that is, normal pregnancy versus spontaneous preterm delivery). Only phylotypes present (one read count) in 25% or more of the samples were considered in the analysis.

Read count data obtained from a longitudinal experiment design are typically modeled using generalized estimation equations (GEE) or linear mixed-effects (LME) models by assuming a Poisson or negative binomial distribution of the response. The choice of a Poisson distribution is justified when the count variance equals the count mean, while the negative binomial distribution is preferred when the mean-variance equality cannot be safely assumed.

Several phylotypes were not detected in a large proportion of samples; hence, the frequency of 0 count values in the dataset was larger than expected under a Poisson or negative binomial distribution. Therefore, models that allow for zero inflation are more appropriate; indeed, this approach has been used for decades [111].

To ensure a proper fit of the count data of each phylotype, we utilized zero-inflated negative binomial mixed-effects models (ZINBLME) in addition to negative binomial linear mixed effects (NBLME) and Poisson linear mixed effects (PLME) models. These three types of models were fitted to each phylotype, and the model with the lowest Akaike Information Criterion (AIC) value was retained. The *P*-value for the association between the microbial relative abundance and the group variable was computed only for the best model (smallest AIC).

The mixed effects modeling of the read counts data (dependent variable) on pregnancy status (independent variable) was performed using the NLMIXED procedure in SAS (version 9.3) as discussed elsewhere [97,112,113]. All three types of models (PLME, NBLME and ZINBLME) included an offset term (the log of the total number of reads in a given sample) to allow for a comparison of the relative abundance (and not absolute counts) between groups. The random effect in the ZINBLME models was allowed only on the non-zero inflation component (negative binomial mean).

For each of the three types of models, the reported coefficient represents the difference in mean log relative abundance between patients who subsequently had a spontaneous preterm delivery and those who delivered at term, which was further converted into a fold change. The *P*-value of the model with the best fit (smallest AIC) was retained and false discovery rate adjustment was applied across the phylotypes. A q-value <0.1 and fold change >1.5 were considered significant.

### Analytical approach to examine changes in abundance of phylotypes with gestational age

The approach used to identify phylotypes associated with spontaneous preterm delivery, described above, was also used to characterize changes in the phylotypes’ abundance as a function of gestational age. The gestational age range over which samples were obtained in this longitudinal study of pregnant women who deliver at term was divided into three intervals: 6.9 to 22.1, 22.2 to 29.8 and 29.9 to 41 weeks. The two cut-off points at 22.1 and 29.8 weeks were selected so that the resulting three intervals had comparable gestational age windows and a comparable number of vaginal samples. The 5th and 95th percentiles of the gestational age over which samples were collected, were calculated. Then, the interval between the 5th and 95th percentile was divided into three gestational age windows (14.5 to 22.1 weeks, 22.2 to 29.8 weeks and 29.9 to 37.5 weeks).

This analysis provides a simple description of the gestational age-related trends in microbial abundance (for example, an increase in abundance of approximately two fold from the first to second interval). However, such an approach may not capture potentially more complex trends in the microbial abundance as a function of gestational age. Therefore, a secondary analysis was performed by treating gestational age as a continuous variable. Orthogonal polynomial terms based on gestational age were used as explanatory variables in a NBLME model. The response variable in this model was the observed number of reads for each phylotype in each sample. The degree of the polynomial function was selected so that the resulting model minimized the AIC criterion. The degree of the polynomial function varied from 1 to 7. The *P*-values for the ‘between intervals’ comparisons as well as the *P*-value for each polynomial term were adjusted across phylotypes. A false discovery rate of 10% was used.

## Results

### Characteristics of the study population

The clinical and demographic characteristics of pregnant women who had a term delivery or a spontaneous preterm delivery are displayed in Table 1. The median number (interquartile range, IQR) of samples for term and preterm deliveries was 4 (2 to 6) and 3 (2 to 4), respectively, for a total of 349 samples. There were no significant differences in the age, race, pre-pregnancy body mass index and nulliparity between the groups (all *P* >0.05). As expected, preterm neonates had lower birthweights and Apgar scores than term neonates (Table 1).

**Table 1:** Clinical and demographic characteristics of the study population

	**Term delivery (n = 72)**	**Spontaneous preterm delivery (n = 18)**	**P value**
Age (years)	24 (21.8-28)	21 (20–26)	0.1
Race:			
African American	62 (86.1%)	17 (94.4%)	0.7
White	4 (5.6%)	1 (5.6%)	
Others	6 (8.3%)	0	
Pre-pregnancy BMI (kg/m^2^)	28.7 (25.7-35.3)*	25.7 (21.6-33.6)**	0.2
Nulliparity	18 (25%)	7 (38.9%)	0.3
Gestational age at delivery (weeks)	39.6 (38.8-40.7)	30.5 (28–33.1)	0.001
Birthweight (grams)	3295 (3124.3-3538.8)	1402.5 (997.5-1998.8)	0.001
Apgar score at 1 min	9 (8–9)	6 (3–8)	0.001
Apgar score at 5 min	9 (9–9)	8 (6–8)	0.001
Duration of hospital stay (neonates); in days	3 (3–3)**	26 (13–53)***	0.001

Data presented as median (interquartile range) or n (%).

BMI: body mass index.

Missing data: *n = 4, **n = 6, ***n = 1.

### Characterization of the microbial taxa as a function of depth of coverage

We characterized the vaginal microbiota using pyrosequencing of barcoded 16S RNA genes. The data set consisted of 2,639,039 high quality sequences, with a median length of 433 bp (IQR: 391 to 475). The median number of sequences per sample was 7,548 (IQR: 5,388 to 9,489). Taxonomic assignment of the individual sequence reads identified a total of 99 taxa in the vaginal microbiota of the women studied; all 99 taxa were observed both in pregnant women who delivered preterm and in those who delivered at term. The taxonomic assignment of vaginal bacterial community members is shown in Additional file 1: Table S1.

### The vaginal microbiota of women who deliver preterm versus those who deliver at term

Our attempt to identify phylotypes with relative abundances that were significantly different between women who delivered at term and those with spontaneous preterm delivery was based on statistical models appropriate for the type of data generated and that: 1) were designed for count data modeling (assuming Poisson and negative binomial distributions); and 2) allowed for correlated observations from the same individual (for example, linear mixed effect models); while 3) allowing for extra zeroes in the data since some phylotypes were frequently undetected. Only phylotypes that were present in at least 25% of all samples were included in the analysis, restricting the number of phylotypes to 21 (Additional file 2: Figure S1).

Table 2 shows the AIC statistics for all three types of models for each phylotype, as well as the estimate, confidence interval and *P*-value for the best (smallest AIC) model. These analyses did not reveal any differences in the relative abundance of bacterial phylotypes between women who delivered preterm and those who delivered at term. In addition, among women who had a spontaneous preterm delivery, we did not find differences in the relative abundance of bacterial phylotypes between women with and without acute histologic chorioamnionitis.

**Table 2:** Phylotypes differential relative abundance between pregnant women who delivered preterm and at term

**Phylotypes**	**PLME AIC**^**a,d**^	**NBLME AIC**^**b,d**^	**ZINBLME AIC**^**c,d**^	**Best AIC**^**d**^	**Estimate**	**Lower 95% CI**	**Upper 95% CI**	**Fold change**	**p-value**	**q-value**^**e**^
**Non-significantly different phylotypes**										
*Prevotella* genogroup 3	6017.7	NA	1160.3	ZINBLME	2.524	0.341	4.706	12.5	0.0239	0.3850
*Dialister* sp. type 2	3934.3	4142.6	1346.3	ZINBLME	1.630	−0.002	3.261	5.1	0.0502	0.3850
*Sneathia sanguinegens*	2875.4	NA	1121.2	ZINBLME	1.510	−0.033	3.053	4.5	0.0550	0.3850
*Parvimonas micra*	4338.8	NA	1242.3	ZINBLME	1.339	−0.458	3.136	3.8	0.1422	0.5972
*Gemella*	4753.1	NA	1296.3	ZINBLME	1.327	−0.225	2.879	3.8	0.0927	0.4867
BVAB2	12246	NA	1686.4	ZINBLME	1.129	−0.784	3.041	3.1	0.2440	0.7046
*Lactobacillus jensenii*	54150	NA	2859	ZINBLME	1.040	−0.518	2.597	2.8	0.1880	0.6580
BVAB1	116789	NA	2467.9	ZINBLME	0.915	−0.976	2.807	2.5	0.3389	0.7046
*Megasphaera* sp. type 1	38178	NA	2860.1	ZINBLME	0.717	−0.864	2.298	2.0	0.3700	0.7046
*Dialister propionicifaciens*	3433.2	4330.8	1529.3	ZINBLME	0.625	−0.738	1.987	1.9	0.3648	0.7046
*Lactobacillus coleohominis*	2309.1	NA	1405.6	ZINBLME	0.550	−0.800	1.899	1.7	0.4206	0.7046
*Gardnerella vaginalis*	193277	166894	4311.4	ZINBLME	0.520	−0.961	2.000	1.7	0.4874	0.7046
*Aerococcus christensenii*	18185	17841	2505.6	ZINBLME	0.383	−1.107	1.872	1.5	0.6108	0.7987
*Atopobium vaginae*	NA	NA	2625.8	ZINBLME	0.376	−1.249	2.001	1.5	0.6466	0.7987
*Lactobacillus crispatus*	159892	125388	3788	ZINBLME	0.157	−1.784	2.098	1.2	0.8724	0.9068
*Lactobacillus iners*	263630	194492	6324.8	ZINBLME	0.045	−0.721	0.811	1.0	0.9068	0.9068
*Eggerthella*	2926.9	NA	1405.3	ZINBLME	−0.189	−1.724	1.345	−1.2	0.8069	0.8918
*Lactobacillus vaginalis*	2682.8	NA	1149.1	ZINBLME	−0.293	−1.940	1.354	−1.3	0.7248	0.8456
*Ureaplasma parvum*	1866.8	NA	1184.9	ZINBLME	−0.374	−1.480	0.732	−1.5	0.5033	0.7046
*Atopobium rimae*	1386.9	NA	883.8	ZINBLME	−0.522	−1.910	0.865	−1.7	0.4567	0.7046
*Lactobacillus gasseri*	34741	NA	1592.4	ZINBLME	−0.864	−2.847	1.118	−2.4	0.3887	0.7046

^a^PLME: Poisson Linear Mixed Effects Model.

^b^NBLME: Negative Binomial Linear Mixed Effects.

^c^ZINBLME: Zero-Inflated Negative Binomial Mixed-Effects Model.

^d^AIC: Akaike Information Criterion.

^e^q-value is p-value after adjustment for false-discovery rate (0.1).

### Dynamic changes in vaginal microbiota as a function of gestational age

To examine whether the vaginal microbiota changes with gestational age, we focused on women with a normal pregnancy who delivered at term (n = 72). We tested this hypothesis by categorizing gestational age into three intervals and also by treating gestational age as a continuous variable in linear mixed-effects models. Based on the analysis in which the gestational age was categorized in three intervals, we found that the relative abundance of four *Lactobacillus* spp. (*L. crispatus*, *L. jensenii*, *L. gasseri* and *L. vaginalis*) increased as a function of gestational age. Indeed, the mean relative abundance in the third interval (29.9 to 41 weeks) was higher than in the first interval (6.9 to 22.1 weeks) of gestation (q-value <0.1) (Additional file 3: Figure S2 and Additional file 4: Table S2). The relative abundance of eleven other bacterial taxa was found to decrease with advancing gestational age. These included: *Eggerthella*, *Parvimonas micra*, *Dialister* spp. type 2, *Gemella*, bacterial vaginosis associated bacteria 1 (BVAB1), BVAB2, *Atopobium vaginae*, *Gardnerella vaginalis*, *Atopobium rimae*, *Sneathia sanguinegens* and *Ureaplasma parvum*. A separate analysis in which gestational age was treated as a continuous variable confirmed all positive findings from the three-interval based approach (Additional file 5: Table S3).

### Vaginal microbial community structures in women who delivered at term and those who had a spontaneous preterm delivery

**Figure 1:**
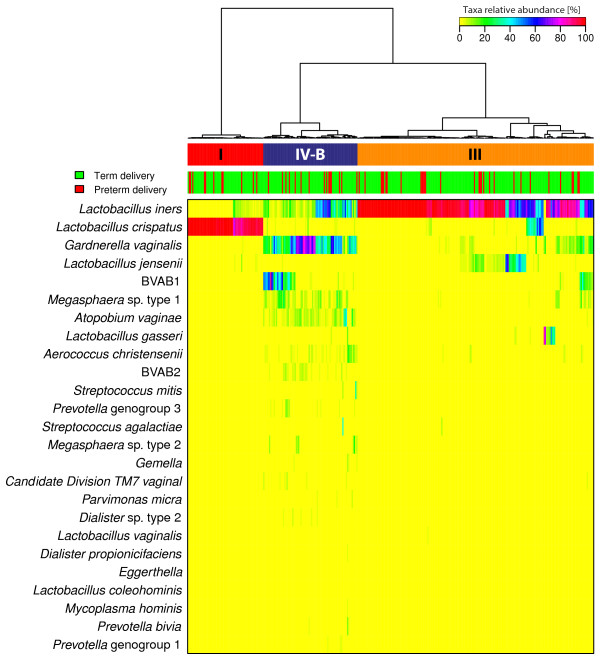
**Heatmap of microbial taxa relative abundance identified in the vaginal microbiota of women who delivered at term and women who delivered preterm.** Ward linkage clustering of samples based on the composition and relative abundance of the 25 most abundant species in the vaginal microbiota that define community state types is shown above the heatmap.

In order to visualize the structure of the microbial community of the vaginal ecosystem in pregnant women who delivered at term versus those who delivered preterm, we hierarchically clustered the vectors of relative abundances of bacterial phylotypes (one per sample) using the Jensen-Shannon metric and Ward linkage [110]. In this study, a ‘community state’ refers to a vector of relative abundances of bacterial phylotypes for a given sample. Community states clustered into three groups with similar bacterial composition and abundance (Figure 1), referred to as community state types (CST), according to the nomenclature established by Gajer *et al*. [110].

**Figure 2:**
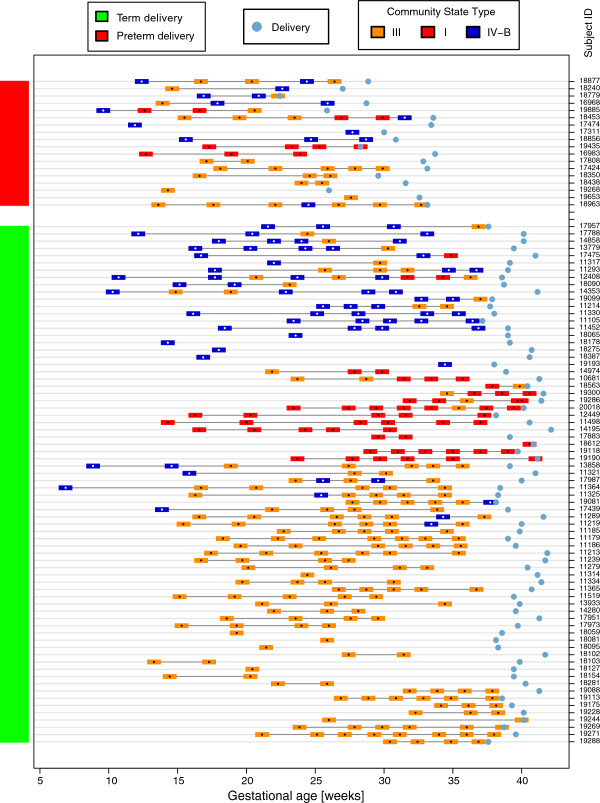
**Profiles of community state types for women who delivered at term and women who delivered preterm as a function of the gestational age.** Each sample in the longitudinal series was assigned to community state types as defined in Figure 1 and is indicated by a rectangle colored according to the legend shown above. Time of delivery is depicted by a light blue circle.

Two of these CSTs were most often dominated by *L. crispatus* (CST I) and *L. iners* (CST III). Communities that clustered in CST IV-B lacked a substantial number of *Lactobacillus* spp. and had higher relative abundance of *G. vaginalis*, BVAB1, *A. vaginae* and *Megasphaera* spp. type 1. These taxa have been previously shown to be associated with bacterial vaginosis [80,83,114]. Overall, frequencies of CST I, CST III and CST IV-B in the entire sample set were 18.6%, 58.5% and 22.9%, respectively. There were no differences in the frequency of the different CSTs (CST I, III, IV-B) between women who delivered at term and those who delivered preterm (CST I: 18.4% versus 19.6%; CST III: 59.4% versus 53.6%; CST IV-B: 22.2% versus 26.8%). Longitudinal profiles of CSTs as a function of gestational age and per subject are shown in Figure 2.

A comparison of microbial diversity (Shannon Diversity Index; SDI) between women who had a spontaneous preterm delivery and those who had a term delivery was performed using a LME model. The SDI values were log-transformed to improve normality of the data. No differences in the microbial diversity were found (term delivery: SDI median 0.38; IQR 0.12 to 1.03; preterm delivery: SDI median 0.39; IQR 0.08 to 1.04).

## Discussion

### Principal findings of the study

The study’s principal findings are as follows: 1) the composition of the vaginal microbiota during normal pregnancy changes as a function of gestational age, with an increase in the relative abundance of four *Lactobacillus* spp., and a decrease in anaerobe or strict-anaerobe microbial species as pregnancy progresses; 2) there were no differences in the relative abundance of microbial phylotypes between women who had a spontaneous preterm delivery and those who delivered at term; and 3) no differences were observed in the frequency of the vaginal CSTs (CST I, III, IV-B) between women who delivered at term or preterm.

### The vaginal microbiota of normal pregnant women

A companion study [97] examined the vaginal microbiota in 22 pregnant women, and compared phylotype abundance and the stability of the microbiota with that of 32 non-pregnant women. The larger sample size of the current study allowed us to demonstrate that there are changes in the vaginal microbiota as a function of gestational age. The abundance of 16 taxa was found to change with the duration of pregnancy; of those, four increased (*L. crispatus*, *L. jensenii*, *L. gasseri* and *L. vaginalis*) and eleven decreased (*Eggerthella*, *P. micra*, *Dialister* spp. type 2, *Gemella*, BVAB1, BVAB2, *A. vaginae*, *G. vaginalis*, *A. rimae*, *S. sanguinegens* and *U. parvum*). An interesting finding is that all of the phylotypes that increased in abundance belonged to the genus *Lactobacillus*, while those that decreased were anaerobes. Of note, *L. iners*, the most prevalent *Lactobacillus* spp. in the vaginal microbiota [95], was among six phylotypes (*Prevotella* genogroup 3, *D. propionicifaciens*, *Megasphaera* spp. type 1, *A. christensenii* and *L. coleohominis*) for which the relative abundance did not change significantly between the two groups of pregnant women (Additional file 3: Figure S2).

These observations are relevant to understanding the changes in the vaginal ecosystem with normal pregnancy. Moreover, it is possible that these temporal changes may be meaningful in assessing health and predisposition to disease states. Comparison of the current results with culture-based studies in pregnancy is difficult because sequence-based techniques allow the comprehensive detection of bacteria and assessment of their abundance, which may not be possible with standard cultivation methods.

### The vaginal microbiota of women at risk for preterm delivery

A compelling body of evidence supports a causal association between intra-amniotic infection and spontaneous preterm delivery [18-24,26,29,33,34,36,115,116]. The organisms found in the amniotic cavity are often similar taxonomically to those found in the lower genital tract of pregnant women as demonstrated by using both cultivation and molecular techniques [19,23,25,40,42,48,54,117-120]. Therefore, an ascending pathway has been proposed to be the most frequent cause of intra-amniotic infection [19,20,38,39,44,45].

During the last three decades, accumulating evidence has suggested that changes in the microbial ecosystem of the lower genital tract, often referred to as bacterial vaginosis, atypical or aerobic vaginitis [63,65,121,122], are risk factors for spontaneous abortion [123-128], spontaneous preterm delivery [41,49,51-54,56,58-63,65,94,128-131], intra-amniotic infection [28,31,32,35,40,132-135], puerperal endometritis [58,129,136-139] and adverse perinatal outcomes [40,49,128,131,140-142]. Even though bacterial vaginosis confers risk for spontaneous preterm delivery, the risk is modest, and most women with this condition will deliver at term. Most of the evidence suggests that treatment of bacterial vaginosis with antimicrobial agents (metronidazole or clindamycin) during pregnancy does not reduce the rate of preterm delivery [143-148], and this has been attributed to an inadequate characterization of the changes in the microbial ecosystem of the lower genital tract in patients who subsequently delivered preterm or to gene-environment interactions in susceptible individuals [60,61,149-151].

### Vaginal microbiota of pregnant women who have a spontaneous preterm delivery

The present study was undertaken to address the question of whether the vaginal microbial composition of women destined to deliver preterm is different from that of women who deliver at term, using sequence-based techniques (16S rRNA gene surveys) and samples collected throughout pregnancy in both groups. Using a thorough statistical approach that was appropriate for longitudinally-collected samples, we did not find any bacterial taxa for which the relative abundance was different in patients who delivered preterm than those who delivered at term.

This study did not identify specific bacteria with an increased or decreased relative abundance that were associated with spontaneous preterm delivery. It is important to note that 61% of patients who had a spontaneous preterm delivery had acute histologic evidence of chorioamnionitis, which is considered an indicator of the ‘amniotic fluid infection syndrome’ [152]. Therefore, by design, this study maximized the likelihood of finding changes in the vaginal microbiome in patients who had a spontaneous preterm delivery.

Although one of every four cases of spontaneous preterm labor is associated with microbiologically-proven intra-amniotic infection, it is unclear whether such patients can be identified by the change in the composition and stability of the vaginal microbiota. Changes may be demonstrable in other biological fluids, such as cervical fluid [153]. Assessment of risk for preterm labor/delivery may also require evaluation of the microbial-host interactions (that is, microbial composition, genotype of the host, and the nature of the cellular or soluble immune response). It is possible that the perturbation of the vaginal microbiome leading to intra-amniotic infection is transient and, therefore, difficult to detect using the sample frequency employed in the current study. Characterization of the vaginal microbiota using 16S rRNA gene sequence analysis, while very informative in identifying differences in composition, does not provide information on the functions of individual bacteria or the community in the vagina. Comparative metagenomic analysis (sequencing and comparing the genes and genomes of microbial communities) might identify vaginal bacteria of the same species but with a different genomic makeup (carry different metabolic or biochemical pathways) that 16S rRNA gene sequence analysis cannot distinguish [154]. In addition, comparative metatranscriptomics (sequencing and comparing the suites of genes expressed by members of microbial communities) might detect functions differentially expressed in women who delivered preterm or at term.

### Strengths and limitations

The major strengths of this study are: 1) its longitudinal nature, which allows characterization of the vaginal microbiota over time, prior to spontaneous preterm birth; 2) the quality of the sequence-based techniques (16S rRNA gene) which reduced bias over other methods, including cultivation techniques; 3) the use of analytical and statistical methods specifically designed for the analysis of longitudinal studies; and 4) the definition of preterm delivery was <34 weeks of gestation, minimizing the potential confounding with patients who delivered near term (<37 weeks). Potential limitations of the study include the sample size. There were 18 patients who had a spontaneous preterm delivery. Yet, this is one of the first studies to address the research question using 16S rRNA gene sequence-based techniques. Our report focuses on relative abundance of different community members because it is difficult to interpret bacterial load quantification data from 16S rRNA gene quantitative PCR analysis. Recently, Nelson *et al*. [155] reported that among women reporting a prior preterm delivery, those with higher levels (absolute abundance) of *Leptotrichia*/*Sneathia species*, BVAB1 and *Mobiluncus spp*. as determined using targeted quantitative PCR, prior to 16 weeks gestation, were significantly more likely to experience a spontaneous preterm delivery. These findings were different from those of Wen *et al*. [156], who found that the presence of *Mycoplasma* in the second semester of pregnancy was associated with increased risks of preterm delivery, while the presence of BVAB3 drastically decreases the risk of preterm delivery (however, this was only the case in African American and Hispanic women, but not in Caucasians). We did not find the relative abundance of these taxa to be associated with the vaginal microbiota of women who delivered preterm. Additional studies on the changes in the vaginal microbiome and spontaneous preterm birth are needed. It would be important to characterize the composition of the vaginal microbiota using indices of relative abundance, as well as the overall bacterial absolute abundance.

Although a 16S rRNA gene-based survey of microbial communities is a powerful tool to characterize the composition of a microbial community, this approach provides limited information about the function and role of the vaginal microbial community in health and disease. The use of a metagenomic and meta-transcriptomics approach would add considerable information to the one presented in this study, as would studying the nature of the host immune, endocrine and metabolic responses associated with changes in microbial composition.

## Conclusions

We report that the composition of the vaginal microbiota during normal pregnancy changed as a function of gestational age, with an increase in the relative abundance of four *Lactobacillus* spp., and decreased in anaerobe or strict-anaerobe microbial species as pregnancy progressed. Differences in the human vaginal microbiota between women who subsequently had a spontaneous preterm delivery and those who delivered at term were not detected.

## Abbreviations

AIC: Akaike Information Criterion; bp: base pair; BVAB: bacterial vaginosis associated bacteria; CST: community state type; GEE: generalized estimation equation; IQR: interquartile range; LME: linear mixed-effects; NBLME: negative binomial linear mixed effect; NIH: National Institutes of Health; PBS: phosphate-buffered saline; PCR: polymerase chain reaction; PLME: Poisson linear mixed effect; PPROM: preterm premature rupture of membranes; rRNA: ribosomal RNA; SDI: Shannon Diversity Index; ZINBLME: zero-inflated negative binomial mixed-effect.

## Competing interests

The authors declare that they have no competing interests.

## Authors’ contributions

RR, SSH, PG, AT and JR conceived the study. RR, SSH, JB, PC, JM and TC performed the clinical sampling and samples management. DWF performed DNA extractions, 16S rRNA gene amplifications and sequencing. DWF, PG and JR processed the sequence data. PG and AT performed the statistical analyses. RR, SSH, PG, AT and JR wrote the manuscript. All authors read and approved the final manuscript.

## Supplementary Material

Additional file 1: Table S1 Taxonomic assignments, relative abundance of 16S rRNA gene sequences per taxa, and metadata. Click here for file

Additional file 2: Figure S1 Relative abundance of all phylotypes present in 25% of all longitudinal samples collected from women who delivered at term (blue) and women who delivered preterm without chorioamnionitis (orange) and with chorioamnionitis (red). The Y-axis represents the percent relative abundance of each taxa in a sample, and the X-axis represents each women. Click here for file

Additional file 3: Figure S2 Changes in phylotype relative abundance as a function of gestational age in women who had a term delivery and evaluated with a three-interval-based analysis. The Y-axis represents the log relative abundance of a given taxa while the x-axis is the gestational age at sampling. Each point represents a sample. The two grey vertical dashed lines define three-intervals of gestation. The solid black line represents the mean relative abundance estimated from the Negative Binomial Linear Mixed Effects model, while the dashed curves represent the 95% confidence interval around the prediction. The arrows at the top of each panel indicate which of the three ‘between-interval’ comparisons was significant. The direction of change, which is marked above each arrow, with the words ‘up’ or ‘down’, indicates the increase/decrease in relative abundance with advancing gestational age from the interval at the left end of the arrow to the interval at the right end of the arrow. A red frame represents phylotypes whose relative abundance significantly increased with gestational age, while a blue frame represents phylotypes whose relative abundance significantly decreased with gestational age. A teal frame represents phylotypes whose relative abundance did not change significantly with gestational age. Click here for file

Additional file 4: Table S2 Phylotypes whose relative abundance changes as a function of increasing gestational age. Results from the three intervals-based analysis. Click here for file

Additional file 5: Table S3 Statistical significance of phylotypes whose relative abundance increased or decreased as a function of gestational age (GA) and evaluated with a three intervals-based analysis of GA or a polynomial-based analysis where GA is treated as a continuous variable. Click here for file
