# Supplementary figures and images for: The vaginal microbiota of pregnant women who subsequently have spontaneous preterm labor and delivery and those with a normal delivery at term
Source: Microbiome. 2014 May 27;2:18. doi: 10.1186/2049-2618-2-18 (PMC4066267; doi:10.1186/2049-2618-2-18)

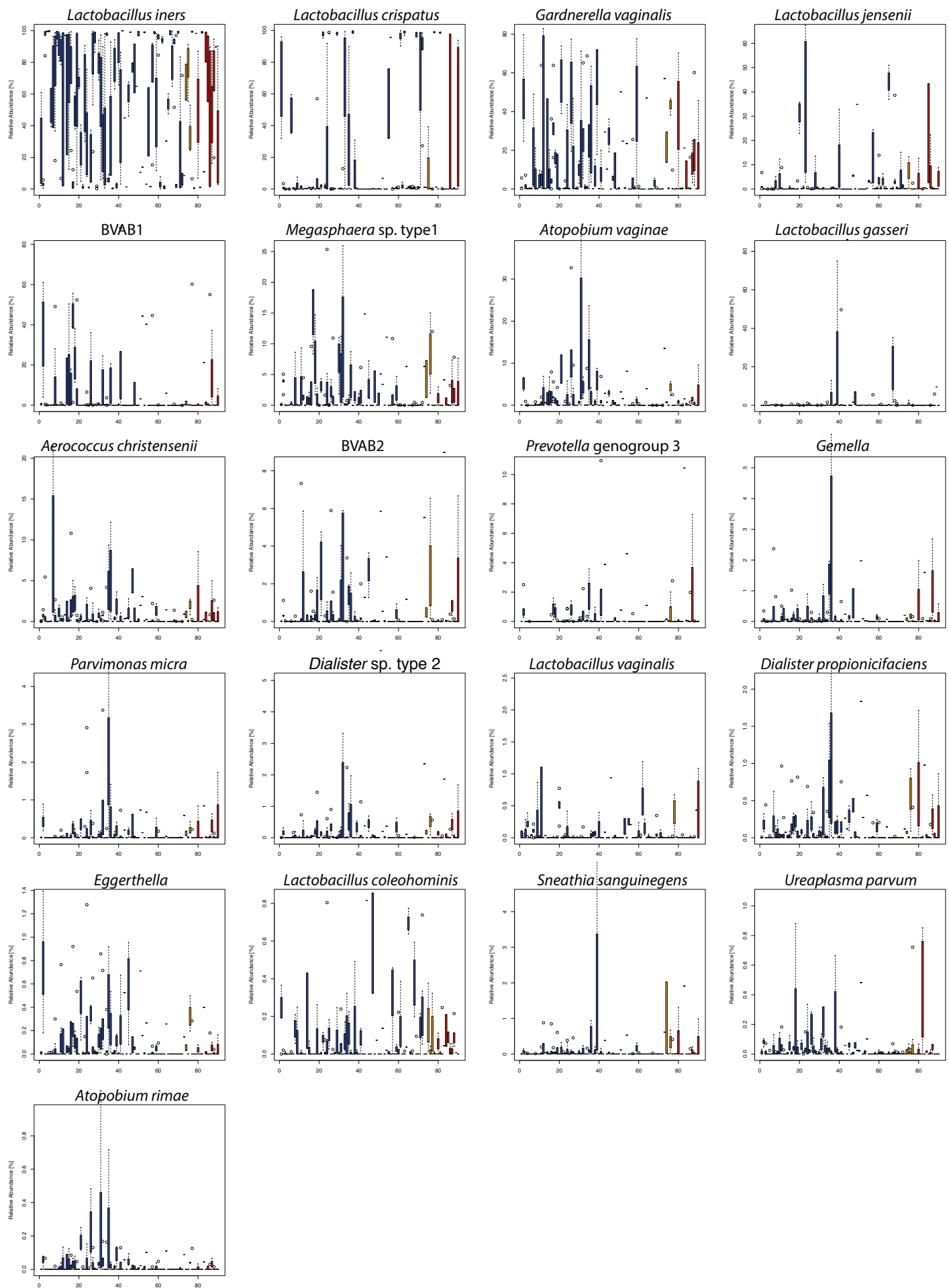

**Figure S1**

Supplement: Additional file 2: Figure S1 — Relative abundance of all phylotypes present in 25% of all longitudinal samples collected from women who delivered at term (blue) and women who delivered preterm without chorioamnionitis (orange) and with chorioamnionitis (red). The Y-axis represents the percent relative abundance of each taxa in a sample, and the X-axis represents each women. [file 2049-2618-2-18-S2.pdf]

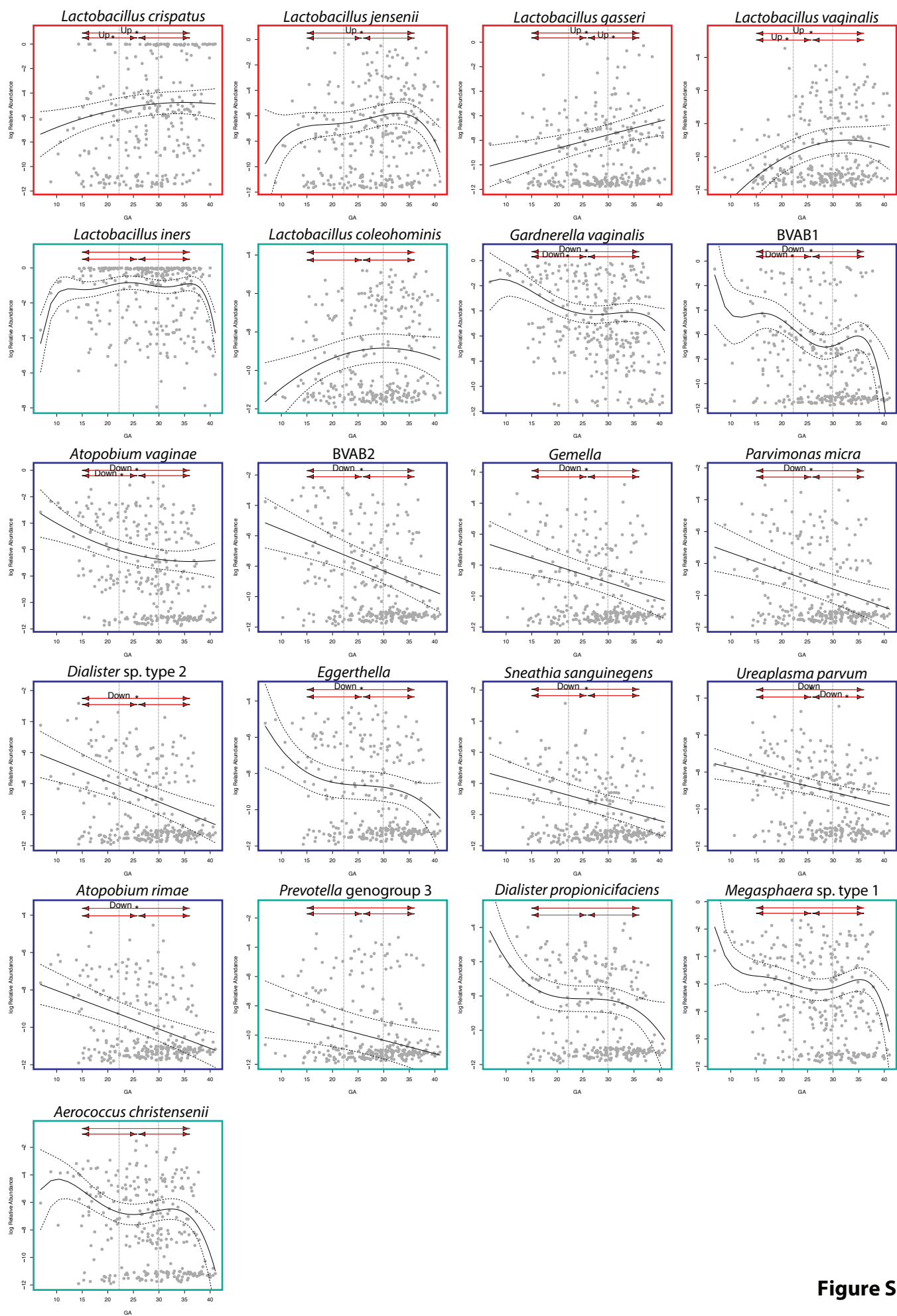

**Figure S2**

Supplement: Additional file 3: Figure S2 — Changes in phylotype relative abundance as a function of gestational age in women who had a term delivery and evaluated with a three-interval-based analysis. The Y-axis represents the log relative abundance of a given taxa while the x-axis is the gestational age at sampling. Each point represents a sample. The two grey vertical dashed lines define three-intervals of gestation. The solid black line represents the mean relative abundance estimated from the Negative Binomial Linear Mixed Effects model, while the dashed curves represent the 95% confidence interval around the prediction. The arrows at the top of each panel indicate which of the three ‘between-interval’ comparisons was significant. The direction of change, which is marked above each arrow, with the words ‘up’ or ‘down’, indicates the increase/decrease in relative abundance with advancing gestational age from the interval at the left end of the arrow to the interval at the right end of the arrow. A red frame represents phylotypes whose relative abundance significantly increased with gestational age, while a blue frame represents phylotypes whose relative abundance significantly decreased with gestational age. A teal frame represents phylotypes whose relative abundance did not change significantly with gestational age. [file 2049-2618-2-18-S3.pdf]
