# Supplementary material for: The vaginal microbiota of pregnant women who subsequently have spontaneous preterm labor and delivery and those with a normal delivery at term
Source: Microbiome. 2014 May 27;2:18. doi: 10.1186/2049-2618-2-18 (PMC4066267; doi:10.1186/2049-2618-2-18)
Supplement: Additional file 4: Table S2 — Phylotypes whose relative abundance changes as a function of increasing gestational age. Results from the three intervals-based analysis. [file 2049-2618-2-18-S4.pdf]

Table S2. Phylotypes whose relative abundance changes as a function of increasing gestational age. Results from a three-interval based analysis.

	GA interval 2 vs GA interval 1						GA interval 3 vs GA interval 1						GA interval 3 vs GA interval 2					
**Phylotypes**	**Estimate**	**Lower 95% CI**	**Upper 95% CI**	**Fold Change**	**p-value**	**q-value**	**Estimate**	**Lower 95% CI**	**Upper 95% CI**	**Fold Change**	**p-value**	**q-value**	**Estimate**	**Lower 95% CI**	**Upper 95% CI**	**Fold Change**	**p-value**	**q-value**
**Phylotypes for which abundance decreases with GA**																		
BVAB1	-2.389	-3.424	-1.354	-10.9	0.0001	0.0021	-2.278	-3.417	-1.139	-9.8	0.0002	0.0042	0.111	-0.767	0.989	1.1	0.8018	0.9462
Gardnerella vaginalis	-0.856	-1.473	-0.240	-2.4	0.0072	0.0378	-0.897	-1.526	-0.267	-2.5	0.0059	0.0131	-0.040	-0.556	0.475	-1.0	0.8764	0.9462
Atopobium vaginae	-0.916	-1.669	-0.162	-2.5	0.0180	0.0756	-1.250	-2.022	-0.479	-3.5	0.0019	0.0057	-0.335	-1.005	0.336	-1.4	0.3227	0.5647
Dialister sp. type 2	-1.145	-2.181	-0.109	-3.1	0.0308	0.1078	-1.744	-2.776	-0.712	-5.7	0.0012	0.0050	-0.599	-1.555	0.357	-1.8	0.2156	0.5009
BVAB2	-1.154	-2.238	-0.069	-3.2	0.0375	0.1125	-2.125	-3.318	-0.932	-8.4	0.0007	0.0047	-0.969	-2.006	0.068	-2.6	0.0665	0.2328
Atopobium rimae	-0.645	-1.406	0.117	-1.9	0.0956	0.1625	-1.473	-2.288	-0.658	-4.4	0.0006	0.0047	-0.828	-1.536	-0.120	-2.3	0.0225	0.1181
Sneathia sanguinegens	-0.748	-1.639	0.143	-2.1	0.0986	0.1625	-1.295	-2.230	-0.359	-3.6	0.0074	0.0131	-0.547	-1.447	0.353	-1.7	0.2297	0.5009
Parvimonas micra	-0.594	-1.623	0.435	-1.8	0.2539	0.3332	-1.410	-2.471	-0.349	-4.1	0.0099	0.0160	-0.816	-1.798	0.166	-2.3	0.1020	0.3060
Ureaplasma parvum	-0.294	-0.826	0.237	-1.3	0.2736	0.3380	-1.015	-1.601	-0.428	-2.8	0.0009	0.0047	-0.721	-1.253	-0.188	-2.1	0.0087	0.0914
Gemella	-0.412	-1.441	0.617	-1.5	0.4271	0.4789	-1.380	-2.482	-0.278	-4.0	0.0148	0.0222	-0.968	-1.976	0.039	-2.6	0.0594	0.2328
Eggerthella	-0.192	-0.937	0.554	-1.2	0.6101	0.6101	-1.017	-1.755	-0.280	-2.8	0.0075	0.0131	-0.826	-1.521	-0.131	-2.3	0.0206	0.1181
**Phylotypes for which abundance increases with GA**																		
Lactobacillus crispatus	0.970	0.345	1.596	2.6	0.0028	0.0294	0.945	0.272	1.617	2.6	0.0066	0.0131	-0.025	-0.573	0.522	-1.0	0.9269	0.9462
Lactobacillus vaginalis	1.368	0.422	2.314	3.9	0.0052	0.0364	1.522	0.541	2.502	4.6	0.0028	0.0074	0.153	-0.539	0.846	1.2	0.6601	0.9390
Lactobacillus jensenii	0.683	-0.053	1.419	2.0	0.0683	0.1625	0.749	-0.059	1.557	2.1	0.0687	0.0962	0.066	-0.564	0.696	1.1	0.8350	0.9462
Lactobacillus gasseri	0.361	-0.822	1.544	1.4	0.5449	0.5721	1.844	0.721	2.966	6.3	0.0016	0.0056	1.483	0.511	2.454	4.4	0.0033	0.0693

GA Interval 1: 6.9-22.1 weeks
GA Interval 2: 22.2-29.8 weeks
GA Interval 3: 29.9-41 weeks
Estimate: the value of the linear mixed-effect model coefficient for the variable defining whether the GA value is in the interval 2 as opposed to interval 1 of gestation. The value of the coefficient represents the difference in the log relative abundance between gestational age interval groups.
CI: Confidence Interval
Fold change: number of times the relative abundance of the phylotype is higher (lower if negative) in the 2nd interval compared to the 1st interval.
q-value: the False Discovery Rate adjusted p-value across all 24 phylotypes that were tested.
