# Supplementary material for: The vaginal microbiota of pregnant women who subsequently have spontaneous preterm labor and delivery and those with a normal delivery at term
Source: Microbiome. 2014 May 27;2:18. doi: 10.1186/2049-2618-2-18 (PMC4066267; doi:10.1186/2049-2618-2-18)
Supplement: Additional file 5: Table S3 — Statistical significance of phylotypes whose relative abundance increased or decreased as a function of gestational age (GA) and evaluated with a three intervals-based analysis of GA or a polynomial-based analysis where GA is treated as a continuous variable. [file 2049-2618-2-18-S5.pdf]

Table S3. Statistical significance of phylotypes whose relative abundance increased or decreased as a function of gestational age (GA) and evaluated with a three intervals-based analysis of GA or a polynomial-based analysis where GA is treated as a continuous variable.

	Statistical significance GA Interval-based analysis	Statistical significance GA as continous variable
**Phylotypes for which abundance decreases with GA**		
BVAB1	Yes	Yes
Gardnerella vaginalis	Yes	Yes
Atopobium vaginae	Yes	Yes
Dialister sp. type 2	Yes	Yes
BVAB2	Yes	Yes
Atopobium rimae	Yes	Yes
Sneathia sanguinegens	Yes	Yes
Parvimonas micra	Yes	Yes
Ureaplasma parvum	Yes	Yes
Gemella	Yes	Yes
Eggerthella	Yes	Yes
**Phylotypes for which abundance increases with GA**		
Lactobacillus crispatus	Yes	Yes
Lactobacillus vaginalis	Yes	Yes
Lactobacillus jensenii	Yes	Yes
Lactobacillus gasseri	Yes	Yes
Lactobacillus iners	No	Yes
Lactobacillus coleohominis	No	Yes
Prevotella genogroup 3	No	Yes
Dialister propionifaciens	No	Yes
Megasphaera sp. type 1	No	No
Aerococcus christensenii	No	Yes
